# Study of the Structural Mechanical Properties of Drainage Canals Rehabilitated by Spraying Method

**DOI:** 10.3390/polym14142781

**Published:** 2022-07-07

**Authors:** Cong Zeng, Chenkun Gong, Fuzhi Wang, Zihao Zhu, Yahong Zhao, Samuel T. Ariaratnam

**Affiliations:** 1 Faculty of Engineering, China University of Geosciences (Wuhan), Wuhan 430074, China; zengcongcug@outlook.com (C.Z.); 18086486825@cug.edu.cn (C.G.); wangfuzhi0328@aliyun.com (F.W.); haozhu@cug.edu.cn (Z.Z.); ta517357276@outlook.com (Y.Z.); 2 School of Sustainable Engineering and the Built Environment, Arizona State University, Tempe, AZ 85259, USA

**Keywords:** canal structure, in-situ spraying, model tests, force characteristics

## Abstract

A large number of drainage pipes and canals in China have been in disrepair for a long time and there have been problems such as leakage and corrosion. In response to these problems, this paper studies a non-excavation technology for repairing the arched canal structure—the in-situ spraying method. To study the influence of the original canal structure on the mechanical characteristics of the lining structure by in-situ spraying and the restraint effect on the lining structure, a field model test with a similar ratio of 1:2 was conducted in the field test pit. By conducting four stages of three-point concentrated load loading tests, the mechanical characteristics of the lining structure were investigated to reveal the influence of the canal structure on the force of the lining structure. The test results show that: the maximum crack width of the newly added lining structure is 0.27 mm and the normal service ultimate bearing capacity of the arched structure repaired by H-70 reaches 150 kN; comparing the loading test and the numerical simulation results, the difference between the two vault displacement results is 4.65% and the results are relatively consistent. The displacement of the bottom of the lining structure is small and the participation of the bottom plate is small, while the displacement of the upper arch structure of the lining is significantly larger than the lateral displacement, indicating that the canal structure can effectively limit the lateral displacement of the newly added lining and that the canal structure is greatly reduced. The bending moment of the lining structure is improved and the restraint effect on the arch foot is more obvious. This paper proposes the use of H-70 to repair arched canal structures by the in-situ spraying method and seeks to prove the feasibility of this method and fill the gap of research in this area. This paper provides the structural design basis and experimental knowledge for the construction of the repair method, which has important practical significance for the pipeline repair project in the future.

## 1. Introduction

**Figure 1:**
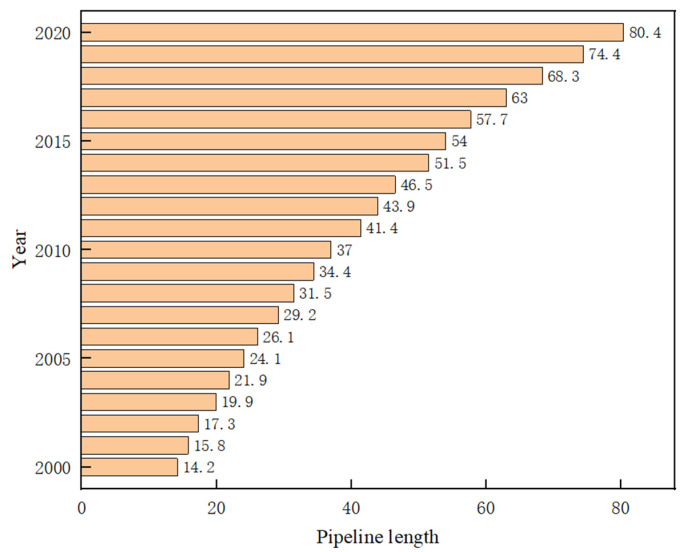
Urban drainage pipeline length [1].

The underground pipeline network is an important infrastructure to ensure the normal operation of a city. As shown in Figure 1, by the end of 2020, the total length of urban drainage pipes in China had reached 80.27 million kilometers [1].

However, with the development of urban drainage pipelines, a large number of in-service pipelines have aged and fallen into disrepair. At present, there are many types of trenchless technologies for pipeline repair, such as cured-in-place pipe [2], slip lining [3], and the stainless steel lining method [4,5]. However, the above methods are suitable for circular pipelines. For the repair of an arched structure, it is most suitable to use the in-situ spraying method. This problem causes varying degrees of structural defects in pipelines, which seriously affects the normal operation of a city. The in-situ spraying method is used for the structural repair of pipelines and is a method of repairing the lining structure by spraying a lining slurry onto the inner wall of pipes. The in-situ spraying method originated from Centriline in the United States in 1933 and is usually called Centriline technology [6]. A section of water supply pipe was repaired by the spraying method in Newark, USA, which improved the flow capacity of the pipe [7]. This technology had become a mature pipeline repair technology by the end of the 20th century [8]. Kong uniformly sprayed high-performance fiber cement mortar on the inner wall of the pipeline and studied the bending resistance of high-performance composite mortar [9]. Wang carried out an experimental study on the durability of engineered cementitious composites (ECC) exposed to sewage environments, simulated by treatments including sulfuric acid exposure and physical erosion [10].The results showed that the residual compressive and tensile strengths of ECC were 30–65% and 300–370% higher than those of the mortar group after 12 corrosion cycles. Wang systematically tested the basic properties of H-70 mortar material [11].

Many scholars have studied the mechanism of lining and the original pipeline. The Water Research Centre studied the influence of mortar layer modulus on the repaired pipeline and found that the deformation of the lining pipe, measured at 8 MPa and 12 MPa, was 0.16% and 0.08%, respectively, while that without lining was 0.56% [12]. Zarghamee and Valentine studied the structural performance of using a prestressed concrete cylinder pipe (PCCP) to repair cracks and found that the PCCP would make contact with the lining at the top and bottom, resulting in large stress in the lining and buckling of the lining [13]. Zhao compared the performance of damaged reinforced concrete pipe (RCP) and sprayed mortar repaired pipe and proposed the calculation method of structural participation strength based on the stiffness reduction weighted area [14]. Some scholars have proposed several models for the coordinated deformation relationship between the lining layer and the pipeline. Wang believes that there is no adhesion between the lining and the pipe and that the lining is equivalent to a new pipe [15]. McAlpine believes that the mortar lining forms a composite structure with the original pipeline and it was found that the lining was mainly tensile and the bending stiffness of the pipeline increased after repair [16]. Zhao and Daigle think that the lining layer and the original pipe form a composite structure and that the interface is completely smooth [17]. The lining thickness also has a great influence on the pipeline performance. To study the influence of the lining wall thickness on the pipeline structure, Cao and Shi found through theoretical analysis that an increase in the wall thickness of the sprayed lining improved the bearing capacity of the pipeline [18,19]. Law and Moore, sampling mortar lining layers, found that if the lining thickness was uneven, with the maximum wall thickness several times larger than the minimum wall thickness, this had a great influence on the lining structure [20]. Najafi and Sever tested the RCP bearing capacity after spraying 0.5-foot and 1-foot mortar repairs and found that a 0.5-foot thick lining increased the bearing capacity by 25% and 1-foot thick lining increased the bearing capacity by 77% [21]. An et al. calculated the flow capacity according to different lining wall thickness formulas and concluded that due to the small friction coefficient of the lining, the flow capacity after repair was not less than the flow capacity of the original pipeline [22].

Most of the above research looked at circular drainage pipelines, but there is less research on drainage canals. The canal structure of the Huangyao River in Wuhan City of China is mainly composed of brick arch culverts, precast concrete circular arches, precast concrete slab culverts, cast-in-situ concrete box culverts, and masonry stone sidewalls or brick sidewalls. The structural defects of the Huangyao River canal are mainly: structural aging, rupture, deformation, leakage, etc. According to CJJ181 [23], some of the canal structure detection is rated as III and IV and the structure may be damaged. It is urgent to carry out reinforcement and reconstruction. At present, the research on canal repair is rare and there is no similar engineering experience for reference. Therefore, this paper proposes an in-situ spraying method to repair the arched canal structure and analyzes the crack development process, structural deformation characteristics, and internal force distribution of lining structures. This paper studies the structural stress after the arched canal structure is repaired and the constraints of properties and channel structures on the lining. This research uses the in-situ spraying method to repair the arched channel body in the actual project and provides the structural design basis and experimental knowledge for the construction of the repair method, which has important practical significance for pipeline repair projects.

## 2. Materials and Test

### 2.1. Raw Materials

The polymer mortar material (H-70) comes from the CUG Trenchless Technology Research Institute in Wuhan, China. The mortar is made using Portland cement as the main gel material and adding reinforcing fibers, quartz sand aggregates and synergistic additives. The material is easy to use and can be stirred with water for 3 min to fluidize. The water/material ratio is 0.15–0.17.

The material test specimens are divided into four types, which are respectively subjected to compression tests, flexural tests, tensile adhesive strength tests, and impermeability tests.

#### 2.1.1. Compression and Flexural Tests

The compressive strength and flexural strength of specimens were tested according to GB/T 17671 [24]. The size of each of the specimens was 160 mm × 40 mm × 40 mm. There were two groups in total, with each group having three specimens, and they were cured in the standard curing box for 24 h and 28 days, respectively. The test loading rate was set to 0.50–0.80 MPa/s and the tensile test loading rate was set to 0.1 MPa/s.

#### 2.1.2. Tensile Adhesive Strength Test

**Figure 2:**
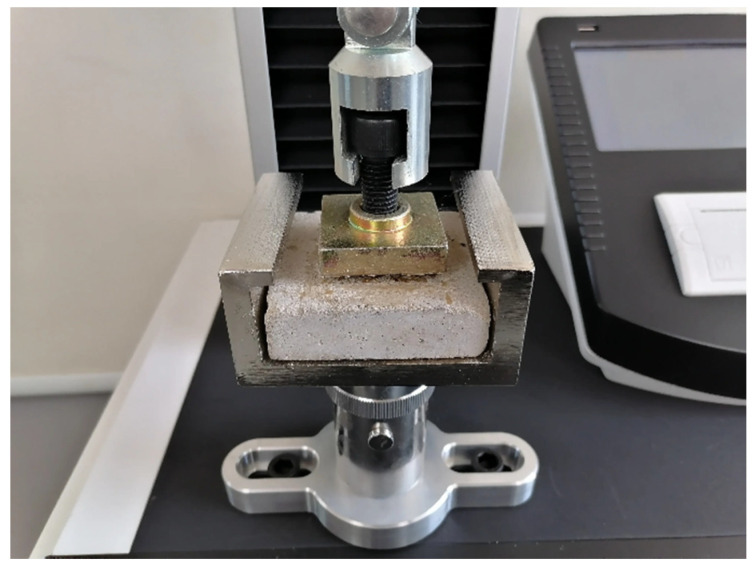
Tensile adhesive strength test.

According to JGJ/T 70 [25], mortar blocks of 70 mm × 70 mm × 6 mm in size were prepared with 10 specimens. The surface of each cured specimen was coated with epoxy resin and the fixture was adhered to the specimen and cured further for 24 h. After the specimen was removal, it was placed on the tensile adhesive strength test machine. Then subject is set to the loading speed of (5 ± 1) mm/min. The test is shown in Figure 2.

#### 2.1.3. Impermeability Test

According to JGJ/T 70, a frustoconical test mold, having a bottom with an upper mouth diameter of 70 mm, a lower mouth diameter of 80 mm, and a height of 30 mm was used. The mixed mortar was loaded into the conical mold and six specimens were made into a total of two groups. After standard curing for 7 days and 28 days, the specimen was packaged into the penetrator with a sealing material for a water permeability test. The test loading rate was 0.1 MPa/h and the impermeability pressure value was recorded.

### 2.2. Design of Test Model

#### 2.2.1. Experimental Modelling

The model was designed according to the similarity theory. Considering the similarity requirements of stress, deformation, and failure mode, the geometric similarity constant was 2 and the similarity constants of stress, strain, linear displacement, and linear load were 2. The similarity constant of elastic modulus and surface load of the model was 1 and the model parameters are shown in Table 1.

#### 2.2.2. Test Site

**Figure 3:**
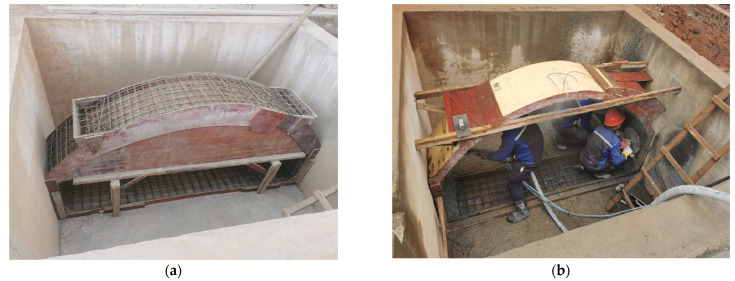
Test model. (**a**) assembling reinforcement; (**b**) spraying concrete.

To approximate the boundary conditions of the actual project, an existing 2500 mm × 2500 mm × 1700 mm test foundation pit on the test floor was used. As is shown in Figure 3, the whole test foundation pit site consists of backfill. The sidewall of the test foundation pit is 240 mm thick sintered ordinary brick and the soil in the local area behind the sidewall at both ends of the model specimen is backfilled twice.

#### 2.2.3. Design of Loading Program

**Figure 4:**
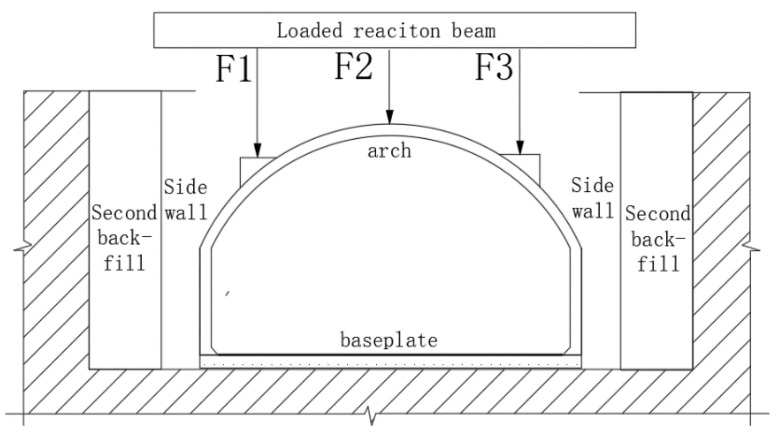
Stress diagram of test model. F1 is the loading point at the arch quarter span; F2 is the loading point at the midpoint of the arch; F3 is the loading point at the three-quarter arch span.

In this experiment, a three-point concentrated load was selected. The stress diagram of the test model is shown in Figure 4. Three concentrated loads were applied to three positions on the upper arch structure by using the jack. The location and size of the concentrated loads were determined according to the principle that the bending moment and axial force of the lining structure are close under the action of uniform load and concentrated load (the vertical resultant force of the two is equal and the left and right loads are symmetrical). The loading ratio of the concentrated loads was F1:F2:F3 = 2:1:2.

**Figure 5:**
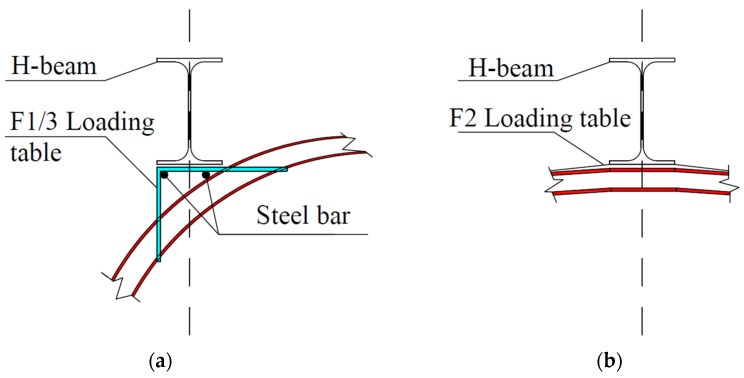
Stress diagram of test model. (**a**) F1/F3 loading table; (**b**) F2 loading table. F1 is the loading point at the arch quarter span; F2 is the loading point at the midpoint of the arch; F3 is the loading point at the three-quarter arch span.

Since the upper arch structure is curved, in order to facilitate loading, a loading table was constructed at the loading point, as shown in Figure 5. To avoid local concrete crushing, additional reinforcement was added to the table and a H-shaped steel distribution beam was set. To ensure full contact between the distribution beam and the loading table, a layer of fine quartz sand was evenly laid on the contact surface.

First, the lining structure was preloaded with three forces acting at the same time, F1 (4 kN, 8 kN), F2 (2 kN, 4 kN), and F3 (4 kN, 8 kN). Considering the load asymmetry and half-span load, F1 is 0.4 F3 under the formal loading, which was divided into four stages. In the first stage, three concentrated forces were divided into 4 levels to load. The initial loads were 4.8 kN, 6.0 kN, and 12.0 kN, respectively. The second stage was divided into seven levels to load and the initial loads of the three forces were 7.2 kN, 4.5 kN, and 0 kN, respectively. The first and second levels of left half-span F1 and F2 were loaded and the initial load was accumulated as an increasing value. After the third level, the full-span loading was restored, with F3 as 18.0 kN and the value was gradually accumulated and then unloaded. The third stage was divided into seven levels to load, where the initial loads of the three forces were 0 kN, 6 kN, and 24 kN, respectively. The first and second right half-span F2 and F3 were loaded and the initial load was accumulated as an increasing value. After the third level, the full-span loading was restored, with F1 as 9.6 kN and this value was gradually accumulated and unloaded. In the fourth stage, the three concentrated forces were loaded in 16 levels at the same time and the initial loads were 9.6 kN, 12.0 kN, and 24.0 kN, respectively. The first three levels were accumulated with the initial load as an increasing value, then accumulated step by step with 2 kN, 2.5 kN, and 5.0 kN, respectively, and then unloaded.

#### 2.2.4. Test Content and Equipment Arrangement

(1) Arrangement of displacement measuring points

**Figure 6:**
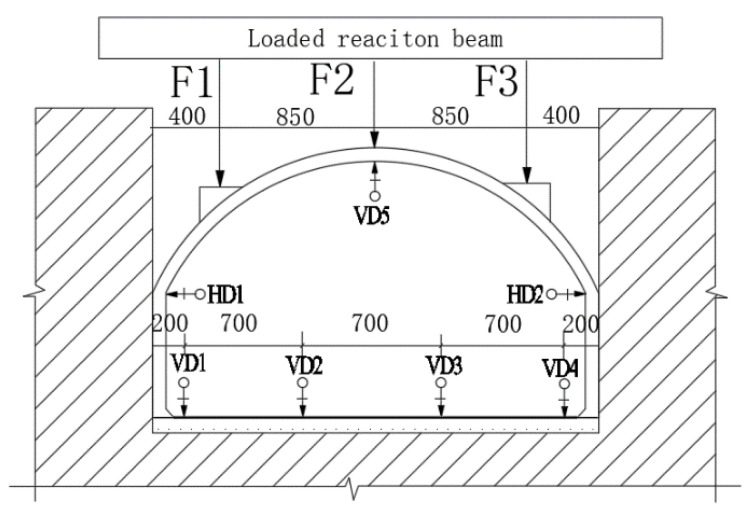
Displacement measuring points. F1 is the loading point at the arch quarter span; F2 is the loading point at the midpoint of the arch; F3 is the loading point at the three-quarter arch span; VD1, VD2, VD3 and VD4 are bottom vertical displacement measuring points; VD5 is the vertical displacement measuring point of the midpoint of the arch; HD1 and HD2 are horizontal displacement measurement points on both sides of the arch foot.

The displacement measuring points were arranged as follows: (1). the vertical displacement of the floor; (2). the horizontal displacement of the side wall top; (3).the vertical displacement of the vault. The specific displacement measuring points are shown in Figure 6. VD and HD represent vertical displacement and horizontal displacement, respectively.

(2) Arrangement of strain measuring points

**Figure 7:**
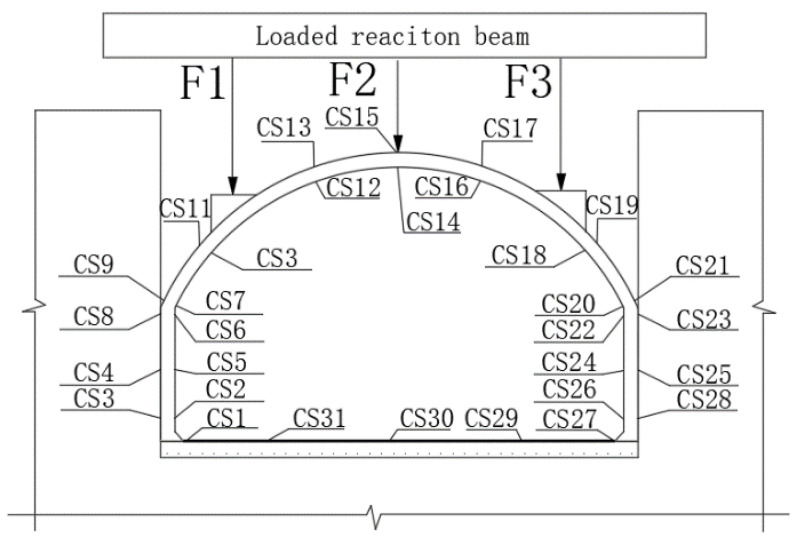
Strain measuring points. F1 is the loading point at the arch quarter span; F2 is the loading point at the midpoint of the arch; F3 is the loading point at the three-quarter arch span; CS1-CS28 are strain measuring points at different positions of the lining structure.

The concrete strain is arranged on the surface of the central line of the plate. The specific measuring points are shown in Figure 7. CS represents the number of measuring points for the concrete strain.

### 2.3. Simulation Process

#### 2.3.1. Material Properties and Meshing

The internal force and deformation of the test model were analyzed based on the Finite Element Software called ABAQUS. The geometric parameters of the model are shown in Table 1. The elastic modulus of the lining structure material was 3.55 × 10^4^ N/mm^2^ and the Poisson’s ratio was 0.2; the elastic modulus of the side wall was 1.8 × 10^3^ N/mm^2^ and the Poisson’s ratio was 0.2.

**Figure 8:**
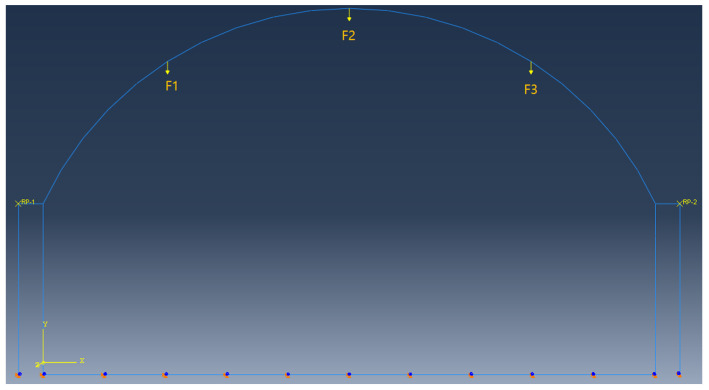
Strain measuring point. F1 is 40 kN; F2 is 50 kN; F3 is 100 kN; RP-1 and RP-2 are the reference points for the contact between the stiff pole and the canal structure.

As shown in Figure 8, the load was defined as a three-point loading method and the corresponding load in the D10 stage of the test was taken. In the D10 stage, F1 was 40 kN, F2 was 50 kN, and F3 was 100 kN. The boundary conditions were set to constrain the degrees of freedom in three directions of the arch and side wall corners and the bottom plate constrained the degrees of freedom in the horizontal direction.

The model adopted B21 beam elements. The upper arch was divided into 36 units, the sides were divided into 24 units, and the base plate was divided into 50 units.

#### 2.3.2. Model Simplification

**Figure 9:**
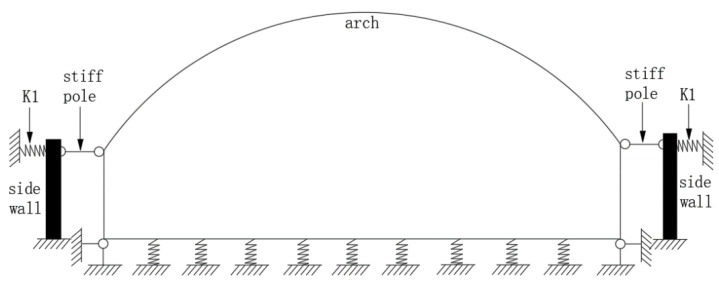
Simplified sketch. K1 is the horizontal soil spring;arch is the The arch is the lined superstructure;side wall is the hypothetical canal structure.

From the displacement measured by the test, it can be seen that the vertical displacement at both ends of the bottom plate was very small, so it was simplified as a hingeless support. The side wall was simplified as a vertical cantilever component and the side wall and the lining structure were connected with rigid rods (only under pressure). The horizontal soil spring K1 was applied to the top of the brick side wall and the vertical soil spring K2 was applied to the middle area of the bottom plate. The calculation diagram is shown in Figure 9.

The soil spring stiffness was calculated according to the proportional coefficient value of the horizontal resistance coefficient of the foundation. The m was taken as 10,000 kN/m^4^. The horizontal soil spring stiffness K1 was approximately 21,250 kN/m and the vertical soil spring stiffness K2 was approximately 2125 kN/m.

## 3. Material Performance and Experimental Results

### 3.1. Material Performance

#### 3.1.1. Compression and Flexural Tests

**Figure 10:**
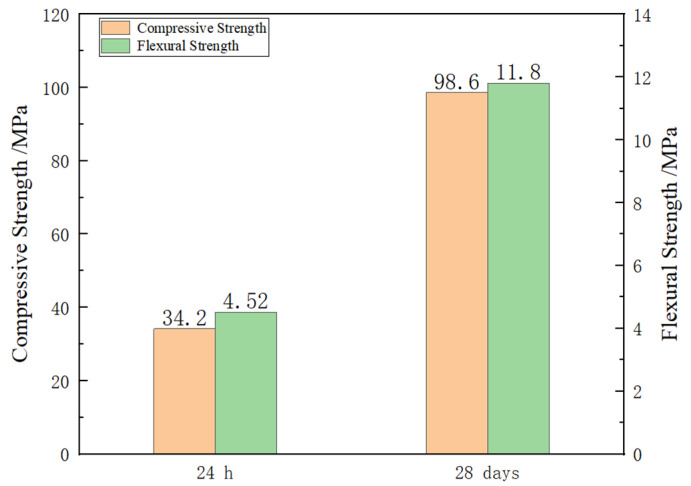
Compressive and Flexural Strength.

The test results of material strength are shown in Figure 10. The compressive strength of the specimen after curing for 24 h was high, indicating that it can be rapidly hardened under standard curing conditions and has high early strength. Due to the addition of reinforced fiber, the specimens reached a high strength level after 28 days. Because the drainage pipeline was in a long-time wet environment and was subjected to the cyclic effect of traffic loads, there was a high requirement for the bending resistance of the pipeline. The material has high early strength and high flexural strength, which can quickly harden and repair under the conditions of the wet inner surface of the pipeline. It has a certain strength to ensure the normal operation of the repaired pipeline in a short time.

#### 3.1.2. Tensile Adhesive Strength Test

The test results are shown in Table 2. The tensile adhesive strength of the material was much higher than that of ordinary mortar and the failure surface appeared in the cement test block. This shows that the material has good bonding performance and can be firmly attached to the inner surface of the pipeline, which is suitable for the repair of drainage pipelines.

#### 3.1.3. Impermeability Test

The test results are shown in Table 3. With the increase in water-cement ratio, the impermeability pressure gradually decreased, indicating that under the same hydration conditions, the increase in water content will lead to more and larger pores in the matrix, thereby reducing the compactness. However, the material still maintained a high osmotic pressure and its matrix was extremely dense and still had a high impermeability, which can greatly extend the service life of the repaired drainage pipeline.

### 3.2. Experimental Results

#### 3.2.1. Test Process and Fracture Description

**Figure 11:**
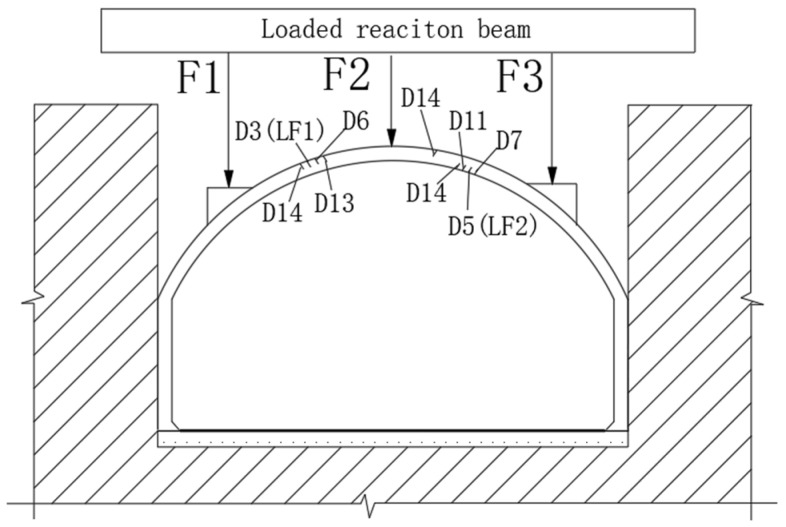
Crack location.

When the fourth stage D3 load was applied, the first micro-crack was found on the top surface of the arch between F1 and F2. The width of the cracks was 0.07 mm, marked as LF1. The maximum width of LF1 was 0.13 mm under the D5 load and a micro crack was found on the inner surface of the arch between F2 and F3. The crack width was 0.02 mm, marked as LF2. When the D6 level load was applied, new cracks appeared near LF1 and LF2 and the original crack width increased. The maximum width was 0.16 mm on LF1. In the process of loading to D11, there were new cracks between F2 and F3. When loading to D14, a micro crack was found on both sides of the arch surface. After unloading, the crack closed well and the relative position of the crack is shown in Figure 11.

From the perspective of the entire crack development, the cracks between F1 and F2 were on the outer surface of the arch and the cracks between F2 and F3 were on the inner surface of the arch in the early stages. Only when the maximum load was D14, did a micro crack appear on the outer surface of the arch, indicating that the bending moment direction of the crack area on both sides was opposite.

During the test, the maximum crack width was 0.27 mm. After unloading, all cracks were well closed and the maximum crack width was 0.02 mm. GB 50152 [26] stipulates that the maximum crack width test allowable value (w_max_) of such components is 0.15 mm. The corresponding load value was between D5 (maximum crack was 0.13 mm) and D6 (maximum crack was 0.16 mm). Without considering the boundary conditions, the load corresponding to the maximum crack width of the original model is 600 kN (the similarity constant is 4).

#### 3.2.2. Displacement Monitoring Results

The displacement of measuring points under various loads is shown in Table 4. The vertical displacement was positive upward and negative downward and the horizontal displacement was positive rightward and negative leftward.

From the above table, it can be seen that the deformation of the bottom plate was very small, the shape rose in the middle and fell at both ends, and the settlement at both ends was very small. The settlement on the right side was greater than that on the left side and the maximum value was 0.04 mm, which is 1/55,000 of the net span of the bottom plate (2200 mm). At the same time, the settlement at both ends increases and then tends to be stable throughout the loading and unloading process. The maximum lateral displacement of the left wall top was 0.36 mm and the maximum lateral displacement of the right wall top was 0.1 mm, which were 1/1944 and 1/7000 of the lateral wall height (700 mm), respectively.

**Figure 12:**
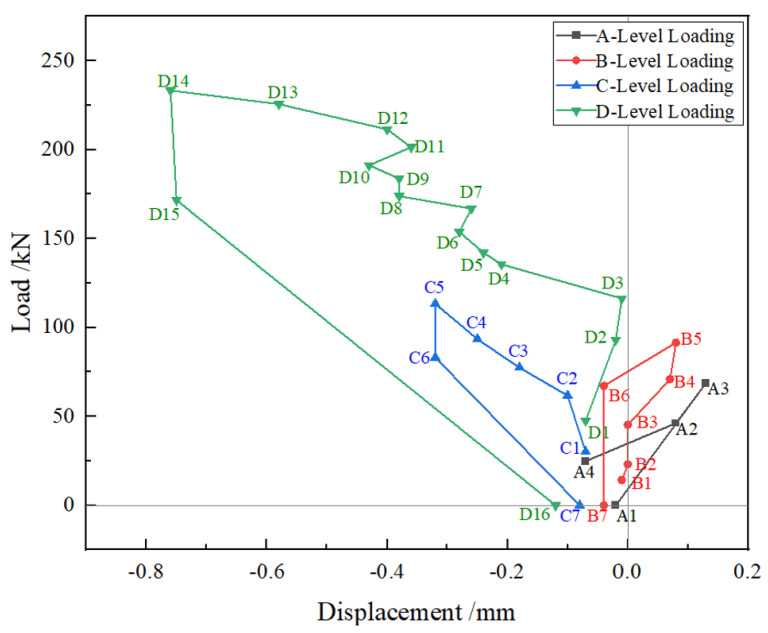
Load-displacement curve of vault.

The load-displacement curve of the vault is shown in Figure 12. The measured vault displacement was mostly positive in the first two stages and negative in the second two stages. Due to the asymmetric load on the left and right sides, the surrounding soil constraints are complex. In the first two stages, the vault moved to the left side significantly and was greater than the vertical deflection. It can be seen from the curve in the figure that during the loading process of the fourth stage, in stages D3, D7, and D11, there was obviously a slow platform, which is consistent with the time of the cracks observed before. The stiffness of the component decreases after the cracks appear. The maximum displacement of vault vertex is 0.76 mm, which is 1/3092 of the net span (2350 mm).

#### 3.2.3. Strain Monitoring Results

**Figure 13:**
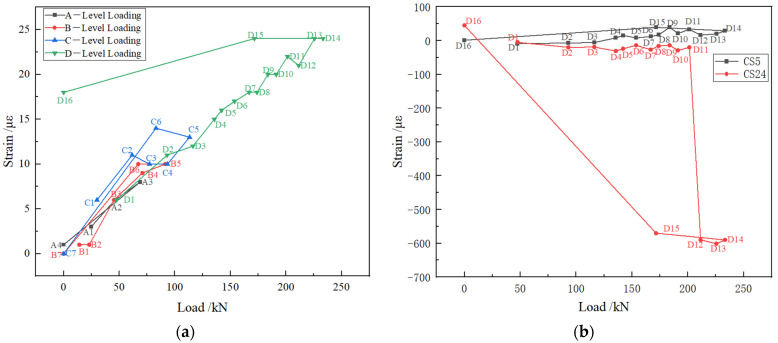
Strain of floor and side wall. (**a**) strain of floor; (**b**) strain of side wall.

From Figure 13, it can be seen that in the four stages of loading process, the trend of the bottom plate strain with the load was basically the same. The overall strain of the bottom plate was small and in the tensile state and the maximum tensile strain was 24 με. Figure 13b shows the variation of the left and right wall strains with the load in the fourth stage. Loading to the fourth stage, the measuring point CS5 on the left wall had always been tensile strain, while the measuring point CS24 on the right wall produced compressive strain and the strains on both sides were similar. When the load reached the D11 stage, the strain at the right wall measuring point increased sharply, reaching the maximum of 610 με.

**Figure 14:**
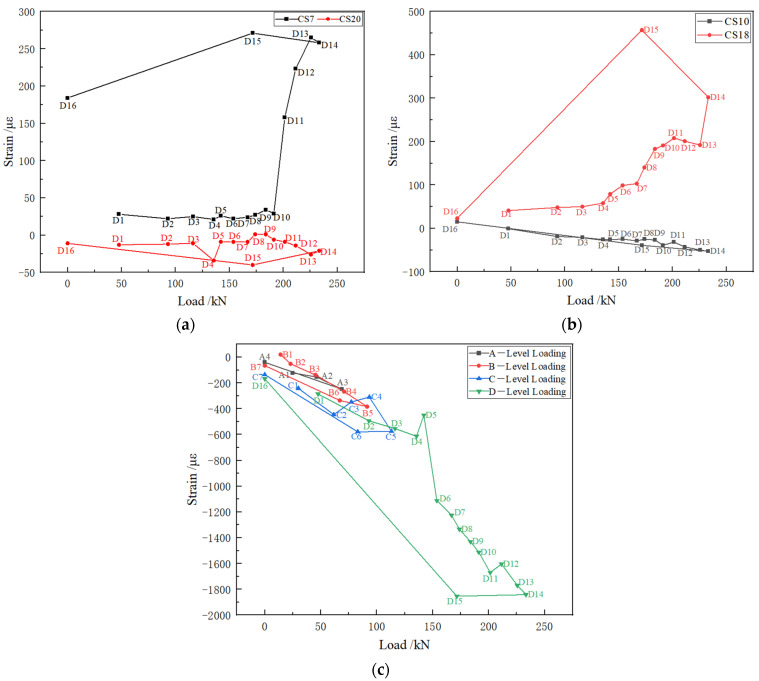
Arch strain curve. (**a**) CS7 and CS20 strains; (**b**) CS10 and CS18 strains; (**c**) CS15 strain.

Figure 14a–c shows the strain changes of the arch toe, arch waist, and vault of the lining structure. It can be seen from Figure 14a that in the early stage of loading in the fourth stage, the strains of the left and right arch feet were similar. The compressive strain appeared on the left side and the tensile strain appeared on the right side. When loaded to the D10 stage, the strain of the right arch toe suddenly increased. After this stage, cracks appeared in the concrete near the arch toe. With the increase in load, the cracks expanded further and the cracks closed after unloading, resulting in this strain trend. As shown in Figure 14b, the strain trend of the arch waist was basically the same as that in Figure 14a. However, after the D4 stage, the strain of the right arch waist increased rapidly due to multiple cracks between F2 and F3. After unloading, the strain decreased greatly, indicating that the cracks near the right arch waist were closed well. Comparing the strain trends of Figure 14a,b, two arch toes and two arch waists, it can be found that the strain of the arch waist is larger than that of the arch toe, indicating that the constraint effect of the sidewall on the arch toe is more obvious.

It can be seen from Figure 14c that the strain trend of the vault was consistent in the four stages of loading. With the increase in load, the compressive strain of the vault increased. When the load reached stage D5, the concrete near the vault cracked and the strain increased sharply. Since no cracks continued to appear near the vault, only the crack propagation stage existed and the strain increase trend decreased.

### 3.3. Simulation Results

**Figure 15:**
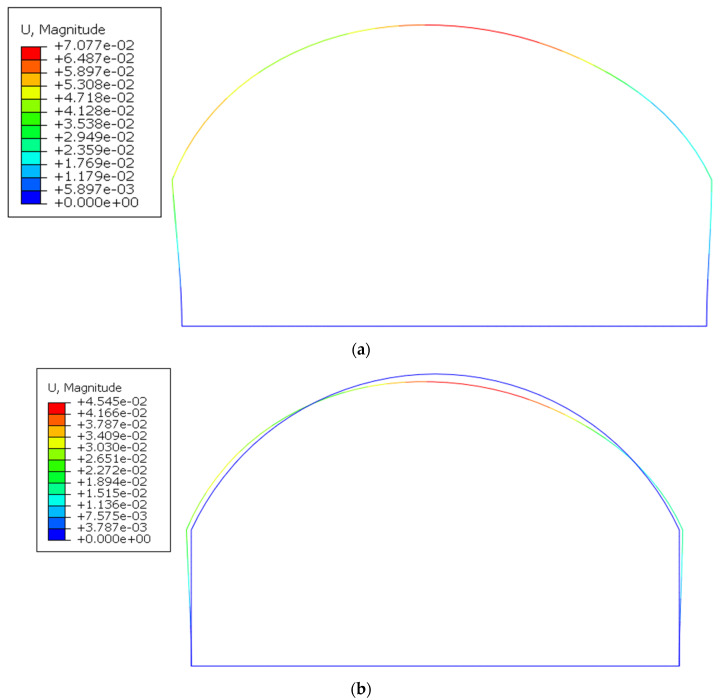
Simulation results of deformations (mm). (**a**) No side wall; (**b**) Side wall.

Under the same other conditions, the influence of the constraint effect of the side walls on the lining structure was compared and analyzed using the D10 load value for calculations. In the absence of side wall constraints, the lining had a large horizontal displacement and the vault displacement was 6.487 mm (Figure 15a). Under the constraint of the side walls, the vault displacement was 4.545 mm (Figure 15b). Compared with the vault displacement (0.43 mm) under the D10 load measured in the experiment in Figure 15, the numerical simulation data were 4.65% different to the experimental data and the two results were relatively consistent. There was a greater limit on the lateral displacement of the lining and the left displacement was greater than the right displacement, which is consistent with the experimental results.

**Figure 16:**
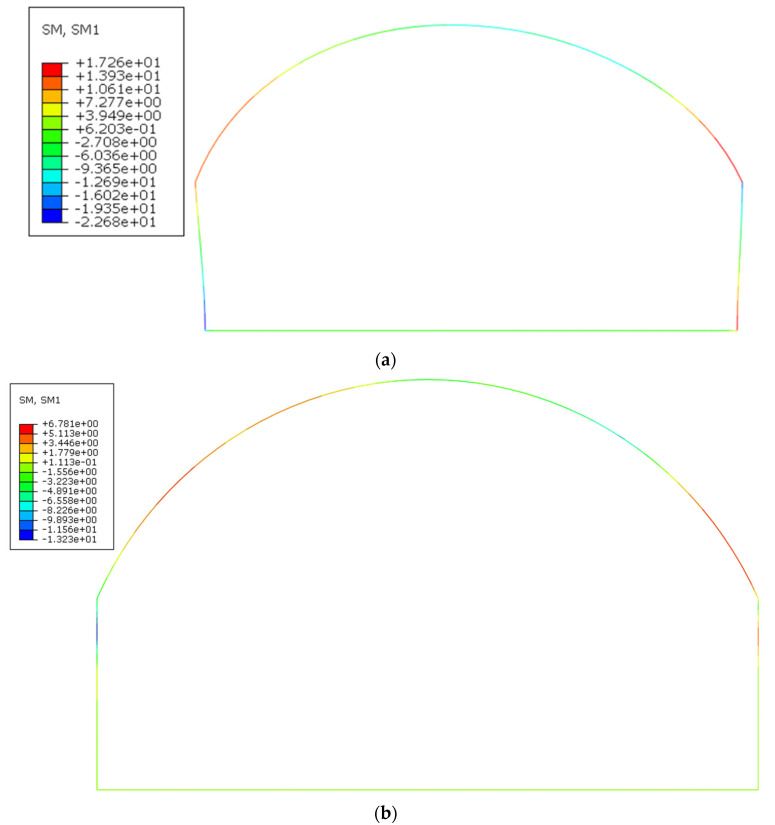
Simulation results of bending moments. (**a**) No side wall; (**b**) Side wall.

Figure 16 shows the bending moment diagram of the lining structure with and without side walls. It can be seen from the figure that the bending moment at the position of F3 was the largest and the bending moment at the outer side between F1 and F2 and the inner side between F2 and F3 was larger. During the test, cracks appeared in these areas. Comparing Figure 16a,b, the bending moment of the vault was reduced from 12.69 kN·m to 4.303 kN·m. The bending moment of the arch foot was reduced from 17.26 kN·m to 4.252 kN·m, which indicated that the side wall had a more obvious constraint effect on the arch foot.

## 4. Conclusions and Future Outlook

At present, there is a lack of research on the mechanical characteristics of lining repair by spraying for arched canal structures and the calculation of this part has not been included in current design specifications. Based on the field model test and finite element simulation, this paper studies the lateral constraint effect of the pre-treated original channel structure on the lining structure. The main conclusions are as follows:

1. H-70 has high early strength, can rapidly harden on the wet pipe surface, and has an extremely dense slurry. It has excellent impermeability and durability and greatly extends the service life of the repaired drainage pipeline. The normal service ultimate bearing capacity of the arched structure repaired by H-70 reaches 150 kN.
2. The first crack on the arch appeared when unilateral stress was applied during the unloading process. During the test, the crack developed slowly, with a maximum crack width of 0.27 mm. After unloading, all the cracks closed well and the maximum crack width was 0.02 mm.
3. The canal structure had a lateral displacement constraint on the lining structure. According to the test results, the strain and settlement of the bottom plate are small and most of the loads are transmitted to the foundation through this combination. The lateral displacement of the vault was significantly greater than the vertical deflection and lateral wall displacement and the left displacement was greater than the right displacement. The strain of the vault and arch waist was greater than that of the arch foot.
4. The lining structure can be simplified as a closed frame without settlement at both ends of the bottom plate. The middle area of the bottom plate and the top of the side wall were loaded with only compressive springs. The comparative test and finite element analysis showed that under the constraint of canal structure, the bending moment of vault and arch foot were greatly reduced and the effect on the arch foot was more obvious.

Comparing the test and the finite element analysis, it was found that the results of the two are relatively consistent. When the canal structure was constrained, the arch foot and the vault of the lining structure were constrained and the lateral displacement was also limited. It has been verified by experiments that it is more suitable to repair the arched canal structure by spraying and that the use of H-70 to repair the canal structure can obtain greater bearing performance. However, this study did not compare the bearing capacity of the independent lining and the original canal structure after repair. The next step can be to study the residual bearing capacity of the canal structure after repair.

## Figures and Tables

**Table 1:** Model parameters.

Serial Number	Physical Parameter		Prototype	Test Model
1	Geometric parameter	Arch span/mm	5000	2500
2		Arch height/mm	1600	800
3		Arch thickness/mm	150	75
4		Side wall height/mm	1400	700
5		Side wall thickness/mm	150	75
6		Floor thickness/mm	150	50
7		Length of member/mm	1000	500
8	Protective layer thickness	Arch/mm	20	10
9		Side wall/mm	20	10
10		Floor/mm	30	15

**Table 2:** Tensile adhesive strength.

Specimen	28 Days Tensile Adhesive Strength /MPa	Failure Interface
Ordinary mortar	0.42	Bond surface
H-70	3.24	Cement test block

**Table 3:** Impermeability test results.

Serial Number	Water/Material Ratio	Permeable Pressure/MPa
1	0.13	2.4
2	0.15	2.2
3	0.17	2.0
4	0.20	1.6

**Table 4:** Displacement of measuring points.

Load Series	Displacement /mm							Remark
	VD1	VD2	VD3	VD4	VD5	HD1	HD2	
A1	−0.01	0.00	0.00	0.00	−0.07	−0.03	0.01	Synchronous loading
A2	−0.01	0.00	0.00	−0.01	0.08	−0.10	0.01	
A3	−0.01	0.00	0.00	−0.01	0.13	−0.11	0.02	
A4	−0.01	0.00	0.00	−0.01	0.02	−0.04	0.01	All unloading
B1	−0.02	0.00	0.00	−0.01	−0.01	−0.05	0.04	F1/F2 Synchronous Loading
B2	−0.02	0.00	0.00	0.00	0.00	−0.05	0.05	
B3	−0.02	0.00	−0.01	−0.01	0.00	−0.06	0.04	F2/F3 Synchronous Loading
B4	−0.01	0.00	−0.01	−0.01	0.07	−0.12	0.04	
B5	0.00	0.01	−0.01	−0.02	0.08	−0.15	0.01	Synchronous loading
B6	−0.01	0.01	−0.01	−0.02	−0.04	−0.15	0.01	F2 Unloading
B7	−0.01	0.00	−0.01	−0.02	−0.04	−0.08	0.02	F1/F3 Unloading
C1	0.00	0.00	−0.01	−0.03	−0.07	−0.12	0.02	F2/F3 loading
C2	0.00	0.00	−0.01	−0.03	−0.10	−0.17	0.03	
C3	−0.01	0.01	0.00	−0.03	−0.18	−0.19	0.02	F1/F2 loading
C4	−0.01	0.01	0.00	−0.03	−0.25	−0.18	0.04	
C5	−0.01	0.00	0.01	−0.03	−0.32	−0.19	0.07	Synchronous loading

## Data Availability

Data are contained within the article.
